# Effect of post-vitrification cryopreservation duration on singleton birth-weight in frozen-thawed blastocysts transfer cycles

**DOI:** 10.3389/fendo.2024.1366360

**Published:** 2024-04-30

**Authors:** Xue Wang, Yaling Xiao, Zhengyi Sun, Wei Xiong

**Affiliations:** Department of Gynecology Endocrine and Reproductive Center, State Key Laboratory of Complex Severe and Rare Disease, Peking Union Medical College Hospital, Chinese Academy of Medical Sciences and Peking Union Medical College, Beijing, China

**Keywords:** birth-weight, blastocyst, cryopreservation duration, singleton, vitrification

## Abstract

**Introduction:**

This study aimed to explore the effect of cryopreservation duration after blastocyst vitrification on the singleton birth-weight of newborns to assess the safety of long-term preservation of frozen–thawed blastocyst transfer (FBT) cycles.

**Methods:**

This was a retrospective observational study conducted at the Gynecological Endocrinology and Assisted Reproduction Center of the Peking Union Medical College Hospital. Patients who gave birth to singletons between January 2006 and December 2021 after undergoing FBT cycles were included. Five groups were formed according to the duration of cryopreservation of embryos at FBT: Group I included 274 patients with a storage time < 3 months. Group II included 607 patients with a storage time of 3–6 months. Group III included 322 patients with a storage time of 6–12 months. Group IV included 190 patients with a storage time of 12–24 months. Group V included 118 patients with a storage time of > 24 months. Neonatal outcomes were compared among the groups. Multivariate linear regression analysis was performed to evaluate birth-weights and other birth-related outcomes.

**Results:**

A total of 1,511 patients were included in the analysis. The longest cryopreservation period was 12 years. The birth-weights of neonates in the five groups were 3344.1 ± 529.3, 3326.1 ± 565.7, 3260.3 ± 584.1, 3349.9 ± 582.7, and 3296.7 ± 491.9 g, respectively (P > 0.05). The incidences of preterm birth, very preterm birth, low birth-weight, and very low birth-weight were similar in all groups (P > 0.05). The large-for-gestational-age and small-for-gestational-age rates did not differ significantly among the groups (P > 0.05). After adjusting for confounding factors that may affect neonatal outcomes, a trend for an increased risk of low birth-weight with prolonged cryopreservation was observed. However, cryopreservation duration and neonatal birth-weight were not significantly correlated (P > 0.05).

**Conclusion:**

The duration of cryopreservation after blastocyst vitrification with an open device for more than 2 years had no significant effect on the birth-weight of FBT singletons; however, attention should be paid to a possible increase in the risk of low birth-weight.

## 1 Introduction

Embryo cryopreservation technology involves the preservation of embryos in an ultra-low-temperature environment (liquid nitrogen, -196°C), followed by thawing to normal physiological temperature when needed. Since clinical pregnancy was first achieved with frozen human embryos in 1983, human embryo cryopreservation has developed rapidly and has become an important part of assisted reproductive technology globally (1). Embryo cryopreservation enables patients to obtain multiple embryo transfer opportunities from a single ovulation induction treatment cycle, maximizing the embryo utilization rate and reducing treatment costs. Embryo cryopreservation improves treatment safety and reduces the incidence of ovarian hyperstimulation syndrome (OHSS) in patients whose fresh cycles cannot be transferred because of endometrial factors or ovarian hyperstimulation (2, 3). Furthermore, embryo cryopreservation provides ample time for biopsy during preimplantation genetic testing (PGT). Additionally, embryo cryopreservation prevents multiple pregnancies by reducing the number of embryos transferred, improving treatment safety and the cumulative pregnancy rate of one oocyte retrieval cycle (4). The cumulative pregnancy rate of several transfer cycles after reducing the number of transferred embryos is not lower than that of a single transfer of multiple embryos, but the former can significantly reduce the multiple pregnancy rate (3, 5, 6). Currently, assisted reproductive centers increasingly promote single-embryo transfers (6). With the improvement of ovarian superovulation protocols and laboratory techniques, as well as the emphasis on fertility protection and the trend of delaying fertility (7), the number of frozen embryos and the duration of embryo cryopreservation have been increasing. This has raised concerns about the influence of cryopreservation duration on pregnancy outcomes and neonatal safety.

The main methods used for embryo cryopreservation are programmed slow freezing and vitrification (8). In the past 10 years, numerous studies have confirmed that embryo survival and clinical pregnancy rates post-vitrification are significantly higher than those after procedural slow freezing; therefore, most centers globally have adopted vitrification for embryo storage (9). Vitrification uses a high concentration of a cryoprotectant to ensure a change from a liquid to a transparent glassy state during rapid cooling, which reduces the freezing damage caused by ice crystals (10). However, whether exposure to high concentrations of dimethyl sulfoxide (DMSO) and ethylene glycol that are used in cryoprotectants have toxic effects on embryos and whether long-term exposure to liquid nitrogen affects the developmental ability of embryos has been controversial (11–13). Some studies have shown that the embryo cryopreservation duration has no adverse effect on embryos’ developmental potential or pregnancy outcomes (14–16), whereas others have found that the cryopreservation duration, particularly that exceeding 6 years, negatively impacts clinical pregnancy and live birth rates (13, 17, 18).

Neonatal birth-weight is considered an indicator of offspring health. Infants born small-for-gestational-age (SGA) or large-for-gestational-age (LGA) are at an increased risk for cardiometabolic abnormalities in childhood and adolescence, increasing the future risk of cardiovascular disease and obesity (19, 20). Therefore, establishing the impact of embryo cryopreservation duration on neonatal outcomes, particularly birth-weight, is important. However, few studies have investigated the effects of storage time after vitrification on neonatal outcomes, particularly on neonatal birth-weight. Several recent retrospective studies have not found a correlation between cryopreservation duration and neonatal outcomes (15, 17, 21); however, the transferred embryos used in some of those studies were mixed, including those at the cleavage and blastocyst stages (15, 17, 21), and pregnancy outcomes differ after cleavage or blastocyst transfer. Several studies have confirmed that clinical pregnancy and live birth rates are higher with blastocyst than with cleaving embryo transfer (22, 23). Therefore, most centers have begun to transfer Day 5 (D5)/6 blastocysts instead of the embryos at cleavage stages. Although blastocyst culture may reduce the number of available embryos transferred, it can eliminate poor-quality embryos, such as those with chromosomal abnormalities, and further screen out embryos with good developmental potential. Therefore, blastocyst cryopreservation and transfer have important clinical application value (23).

In this study, we aimed to evaluate the effect of post-blastocyst vitrification cryopreservation duration, based on the first frozen–thawed blastocyst transfer (FBT) cycle after the oocyte retrieval cycle, on the neonatal birth-weight of singletons, with a view to obtaining data related to the safety of long-term preservation of blastocysts.

## 2 Materials and methods

### 2.1 Study population and design

Since 2005, all embryos at our center have been frozen by vitrified method. Therefore, this retrospective study collected data from patients who underwent an FBT cycle and gave birth to singletons in the Gynecological Endocrinology and Assisted Reproduction Center of Peking Union Medical College Hospital from January 2006 to December 2021. This study was approved by the Ethics Committee of the Peking Union Medical College Hospital. The requirement for obtaining informed patient consent was waived. In this study, each participant was included only once. Women who received egg donation or sperm donation cycles; women with uterine malformations and other related abnormalities; women with some chronic disease, such as thyroid dysfunction; women with pregnancy-induced hypertension and gestational diabetes mellitus; women with abnormal BMI; women with vanishing twin syndrome; women with a history of tumors such as endometrial cancer, or those who underwent PGT, were excluded.

Finally, 1,511 freeze–thaw cycles with single live births were included. Five groups were formed based on the preservation duration of the embryos when performing FBT cycles: Group I included 274 patients with a storage time < 3 months; Group II 607 patients with a storage time of 3–6 months; Group III 322 patients with a storage time of 6–12 months; Group IV 190 patients with a storage time of 12–24 months, and Group V 118 patients with a storage time > 24 months.

### 2.2 Treatment procedure

All treatment procedures were performed according to our center’s routine. The oocytes were retrieved under the guidance of transvaginal ultrasound. Routine *in vitro* fertilization or intracytoplasmic sperm injection was performed based on the quality of the semen. Fertilization was observed on the first-day post-fertilization. Cleaved embryo morphology was observed, and 1–2 cleaved embryos of the best quality were selected for transfer on the third day post-fertilization. At our center, all embryos after fresh cleavage embryo transfer were cultured to D5/6 for blastocyst formation. The Gardner scoring method was used for evaluating blastocysts (24). The cryopreservation criteria for blastocysts were as follows: the blastocyst cavity had expanded to stage 4, and blastocysts with an inner cell mass achieved grade B and above. Blastocysts with an inner cell mass and trophoblast cells of grades B and above were defined as high-quality blastocysts.

### 2.3 Blastocyst vitrification and thawing procedures

All blastocysts were artificially shrunk using a laser system before being frozen. The vitrification freezing medium was divided into three gradients. The first was the basal medium (G-MOPS Plus, Vitrolife, Västra Frölunda, Sweden), in which the shrunken blastocysts were equilibrated for 1–2 min. Next, they were equilibrated for 2 min in the second pre-equilibration medium containing 7.5% (V/V) DMSO and 7.5% (V/V) ethylene glycol. Thereafter, blastocysts were transferred into the third vitrification solution (15% [V/V] DMSO + 15% [V/V] ethylene glycol + 0.65 mol/L sucrose + 10 mg/mL Ficoll), with repeated gentle pipetting, equilibrated for 25–30 s. All operations were performed at 37°C. Blastocysts were then transferred to a cryotop (Kato, Japan) and immersed in liquid nitrogen at -196°C for long-term storage. The liquid nitrogen tank was refilled weekly.

The blastocysts were thawed on the morning of the day of transfer. The thawing procedure was as follows: first, the blastocysts were placed into the basic culture solution and a 0.33 mol/L sucrose solution for 2 min for thawing and then transferred into a second thawing solution, which included basal medium and 0.2 mol/L sucrose solution, for 3 min. Finally, they were placed into the basic culture medium for 5 min, and laser-assisted hatching was conducted. Next, the blastocysts were incubated in a blastocyst culture medium (G2; Vitrolife) for 2 h. All resuscitation operations were performed at 37°C. All freeze–thaw procedures were performed by an experienced embryologist. The freezing procedure and freezing protocols did not change during the study.

### 2.4 Endometrial preparation

Patients underwent endometrial preparation using two approaches (25). Natural cycles were recommended in women with regular ovulation and menstruation. On the 14th day of menstruation, monitored by ultrasound, 2000 U HCG (Ezer, Merck Serono, Darmstadt, Germany) was injected before ovulation, progesterone (Zhejiang Xianchen, Hangzhou, China) was injected intramuscularly at 20–40 mg/day for corpus luteum support, and blastocysts were transferred on the 6th day after ovulation. The artificial cycle method was recommended for patients with irregular menstruation or rare ovulation. Briefly, estradiol valerate (Progynova, Schering, Berlin, Germany) was administered orally from the 2nd to 4th day of menstruation. When endometrial thickness ≥ 8 mm on ultrasound monitoring, the women were injected progesterone intramuscularly. Six days later, the frozen-thawed blastocysts were transferred. One or two blastocysts were transferred once. After transfer, progesterone was injected intramuscularly, or vaginal progesterone gel was used for luteal support.

### 2.5 Measurement index

The primary outcome was the birth-weight of singletons born alive. Other birth outcomes were also assessed. A live birth was defined as birth after 24 weeks of gestation. When calculating the gestational age (GA) of the frozen–thawed blastocysts, the day of transfer was counted as day 19 of the menstrual cycle (26). Normal birth-weight was 2500–4000 g; low birth-weight (LBW) was defined as that < 2500 g, and very low birth-weight (VLBW) as < 1500 g. High birth weight (HBW) was defined as a birth-weight ≥ 4 000 g. Preterm birth (PTB) was defined as delivery at GA 28–37 weeks. Very preterm birth (VPTB) was defined as GA < 32 weeks. SGA was defined as the 10th percentile of birth-weight below the mean weight for GA. LGA was defined as a birth-weight above the 90th percentile of the mean weight for the same GA.

### 2.6 Statistical analysis

Continuous variables are expressed as mean ± standard deviation, and differences among groups were analyzed using a one-way analysis of variance. Categorical variables are expressed as rates (%). Differences among groups were tested using the chi-square or Fisher’s exact test. Multiple regression analysis was used to analyze the effect of blastocyst cryopreservation duration on neonatal outcomes. After adjusting for possible confounders, the odds ratios (ORs), 95% confidence intervals (CIs), and adjusted ORs were calculated. The relationships among cryopreservation duration, birth-weight, and GA were analyzed using multiple linear regression analysis. SPSS software (version 22.0; IBM, Armonk, NY, USA) was used for statistical analysis. Differences were considered statistically significant at P < 0.05.

## 3 Results

Overall, 1,511 patients met the inclusion criteria. **Table 1** shows the patients’ basic data. The five groups included 274, 607, 322, 190, and 118 patients, respectively, with a maximum cryopreservation duration of approximately 12 years. The age of patients at FBT gradually increased. Women with the longest cryopreservation period was the oldest; however, they were the youngest when they underwent the oocyte retrieval procedure (P < 0.05). The duration of infertility increased significantly with increasing cryopreservation duration (P < 0.05). Body mass index (BMI), parity, fertilization method, endometrial preparation protocol, and endometrial thickness did not differ among groups (P > 0.05, respectively), but more women received regular IVF for fertilization in Group IV. The causes of infertility among the groups differed significantly, with the proportion of abnormal ovulation being the highest in Group I (P < 0.05) and the incidence of tubal factors being significantly lower than the other four groups (P < 0.05). The number of blastocysts transferred, and rate of high-quality blastocysts transferred differed significantly among the groups (P < 0.05). Women with the shortest and longest cryopreservation periods were more likely to transfer one blastocyst, and the proportion of good-quality blastocysts transferred was relatively lower in Group III. The proportion of D5 blastocysts transferred differed significantly among the groups, with women with the shortest cryopreservation period having the highest proportion of D5 blastocysts (P < 0.05).

**Table 1:** Comparison of patient baseline data between groups with different cryopreservation duration.

	Group I	Group II	Group III	Group IV	Group V	P	P_a_	P_b_	P_c_	P_d_
N	274	607	322	190	118					
**Cryopreservation duration**	67.9 ± 16.4	126.3 ± 25.4	254.3 ± 52.0	594.3 ± 203.4	1773.7 ± 680.1					
**Age at FBT**	33.3 ± 3.8	33.1 ± 3.9	33.7 ± 4.0	34.9 ± 3.6	36.3 ± 3.9	<0.05	n.s.	n.s.	<0.05	<0.05
**Age at oocyte retrieval**	33.1 ± 3.8	32.7 ± 3.9	33.0 ± 4.0	33.2 ± 3.6	31.5 ± 3.8	<0.05	n.s.	n.s.	n.s.	<0.05
**BMI (kg/m^2^)**	20.5 ± 1.8	20.6 ± 1.7	20.5 ± 1.7	20.4 ± 1.7	20.4 ± 1.7	n.s.	n.s.	n.s.	n.s.	n.s.
**Infertility duration (years)**	3.9 ± 2.6	4.4 ± 2.8	4.4 ± 2.7	5.0 ± 3.1	5.6 ± 3.3	<0.05	<0.05	<0.05	<0.05	<0.05
Type of infertility										
Primary (%)	178 (65.0)	359 (59.1)	189 (58.7)	103 (54.2)	69 (58.5)	n.s.	n.s.	n.s.	<0.05	n.s.
Parity, n (%)										
0	248 (90.5)	541 (89.1)	278 (86.3)	164 (86.3)	101 (85.6)	n.s.	n.s.	n.s.	n.s.	n.s.
> 0	26 (9.5)	66 (10.9)	44 (13.7)	26 (13.7)	17 (14.4)					
Infertility aetiology										
Tubal factors	81 (29.6)	226 (37.2)	123 (38.2)	79 (41.6)	44 (37.3)	n.s.	<0.05	<0.05	<0.05	<0.05
Abnormal ovulation	87 (31.8)	128 (21.1)	72 (22.4)	49 (25.8)	25 (21.2)	<0.05	<0.05	<0.05	n.s.	<0.05
Endometriosis	51 (18.6)	134 (22.1)	78 (24.2)	41 (21.6)	14 (11.9)	n.s.	n.s.	n.s.	n.s.	n.s.
Male factors	70 (25.5)	142 (23.4)	65 (20.2)	40 (21.1)	28 (23.7)	n.s.	n.s.	n.s.	n.s.	n.s.
Others	12 (4.4)	25 (4.1)	7 (2.2)	3 (1.6)	4 (3.4)	n.s.	n.s.	n.s.	n.s.	n.s.
Ovulation stimulation regimen										
GnRHa (%)	169 (61.7)	458 (75.5)	232 (72.0)	154 (81.1)	96 (81.4)	<0.05	<0.05	<0.05	<0.05	<0.05
Fertilization method										
IVF(%)	173 (63.1)	404 (66.6)	213 (66.1)	138 (72.6)	71 (60.2)	n.s.	n.s.	n.s.	<0.05	n.s.
No of blastocysts transferred										
1	136 (49.6)	148 (24.4)	103 (32.0)	52 (27.4)	49 (41.5)	<0.05	<0.05	<0.05	<0.05	n.s.
2	138 (50.4)	459 (75.6)	219 (68.0)	138 (72.6)	69 (58.5)					
**Proportion of good-quality blastocysts transferred**	312 (75.7)	791 (74.2)	376 (69.5)	232 (70.7)	144 (77.0)	<0.05	n.s.	<0.05	n.s.	n.s.
**Proportion of D5 blastocyst transferred**	269/412 (65.3)	628/1066 (58.9)	304/541 (56.2)	199/328 (60.7)	103/187 (55.1)	<0.05	<0.05	<0.05	n.s.	<0.05
Endometrial preparation										
Natural cycle	78 (28.5)	160 (26.4)	70 (21.7)	39 (20.5)	33 (28.0)	n.s.	n.s.	n.s.	n.s.	n.s.
Artificial cycle	196 (71.5)	447 (73.6)	252 (78.3)	151 (79.5)	85 (72.0)					
**Endometrial thickness (mm)**	10.5 ± 1.0	10.6 ± 1.1	10.5 ± 1.1	10.5 ± 1.1	10.6 ± 1.1	n.s.	n.s.	n.s.	n.s.	n.s.

Group I as reference, P_a_: Group II vs. Group I, P_b_: Group III vs. Group I, P_c_: Group IV vs. Group I, Pd: Group V vs. Group I. Bonferroni test was used for comparison between groups. n.s., not signiﬁcant. BMI, body mass index; IVF, in vitro fertilization; ICSI, intracytoplasmic sperm injection; GnRHa, gonadotropin-releasing hormone agonist; GnRHan, gonadotropin-releasing hormone antagonist; FBT, freeze–thawed blastocyst transfer; P < 0.05 is considered statistically significant.

Neonatal outcomes were compared among the different blastocyst vitrification-cryopreservation period groups (**Table 2**). The main outcome was neonatal birth-weight. We found that neonatal birth-weight did not differ significantly (3344.1 ± 529.3, 3326.1 ± 565.7, 3260.3 ± 584.1, 3349.9 ± 582.7, and 3296.7 ± 491.9 g for Groups I, II, III, IV, and V, respectively, P > 0.05). The gestational age was significantly higher in Group I than in the other four groups (P < 0.05, respectively). No significant difference in the incidences of LBW, VLBW, PTB, VPTB, LGA, and SGA was observed in all the groups (P > 0.05, respectively), but we found that the rates of PTB and LBW were higher in Group II, III, IV than in Group I, respectively (P < 0.05). However, the incidence of HBW differed significantly among the five groups (P < 0.05): the HWB incidence was lower in Groups III and V than in the other three groups (P < 0.05). The delivery mode differed significantly among groups, with Group I having the lowest rate of caesarean sections (P < 0.05). No significant difference was observed in the proportion of male babies born between the five groups (P > 0.05).

**Table 2:** Comparison of birth outcomes in newborns in different blastocyst cryopreservation time groups.

	Group I	Group II	Group III	Group IV	Group V	P	P_a_	P_b_	P_c_	P_d_
No	274	607	322	190	118					
Mode of delivery										
Caesarean section (%)	175 (63.9)	434 (71.5)	227 (70.5)	146 (76.8)	79 (66.9)	<0.05	<0.05	n.s.	<0.05	n.s.
Offspring sex										
Male (%)	151 (55.1)	339 (55.8)	155 (48.1)	101 (53.2)	64 (54.2)	n.s.	n.s.	n.s.	n.s.	n.s.
**Birth-weight (g)**	3344.1 ± 529.3	3326.1 ± 565.7	3260.3 ± 584.1	3349.9 ± 582.7	3296.7 ± 491.9	n.s.	n.s.	n.s.	n.s.	n.s.
**Gestational age(d)**	273.2 ± 12.7	270.7 ± 13.9	270.4 ± 15.5	270.0 ± 11.9	270.0 ± 13.7	0.055	<0.05	<0.05	<0.05	<0.05
**PTB**	19 (6.9)	68 (11.2)	38 (11.8)	24 (12.6)	11 (9.3)	n.s.	<0.05	<0.05	<0.05	n.s.
**VPTB**	4 (1.5)	12 (2.0)	7 (2.2)	1 (0.5)	2 (1.7)	n.s.	n.s.	n.s.	n.s.	n.s.
**LBW**	9 (3.3)	38 (6.3)	24 (7.5)	16 (8.4)	9 (7.6)	n.s.	n.s.	<0.05	<0.05	n.s.
**VLBW**	2 (0.7)	3 (0.5)	6 (1.9)	1 (0.5)	1 (0.8)	n.s.	n.s.	n.s.	n.s.	n.s.
**HBW**	30 (10.9)	69 (11.4)	18 (5.6)	22 (11.6)	5 (4.2)	<0.05	n.s.	<0.05	n.s.	<0.05
**LGA**	66 (24.1)	161 (26.5)	71 (22.0)	59 (31.1)	28 (23.7)	n.s.	n.s.	n.s.	n.s.	n.s.
**SGA**	17 (6.2)	20 (3.3)	14 (4.3)	6 (3.2)	5 (4.2)	n.s.	n.s.	n.s.	n.s.	n.s.

Group I as reference, P_a_: Group II vs. Group I, P_b_: Group III vs. Group I, P_c_: Group IV vs. Group I, P_d_: Group V vs. Group I. Bonferroni test was used for comparison between groups. LBW, low birth-weight; VLBW, very low birth-weight; HBW, high birth-weight; PTB, preterm birth; VPTB, very preterm birth; LGA, large-for-gestational-age; SGA, small-for-gestational-age; n.s., not signiﬁcant. P < 0.05 is considered statistically significant.

The relationship between the cryopreservation period of blastocyst vitrification and neonatal outcomes was analyzed using multiple logistic regression analysis, with Group I as the reference group, as shown in **Table 3**. After adjusting for confounding factors that could affect neonatal outcomes, including maternal age, BMI, age at oocyte retrieval, duration of infertility, causes of infertility, parity, fertilization methods, protocols for endometrial preparation, endometrial thickness, quality and quantity of blastocysts transferred, blastocyst development speed, and male baby ratio, the risks for SGA and LGA were similar among the groups (P > 0.05, respectively). However, the risk of LBW was found to be significantly higher in groups III, IV, and V than in group I (adjusted ORs 2.376, 3.147, and 6.593, respectively). The risk of HBW was significantly lower in Group III than in Group I (adjusted OR 0.429, P < 0.05). This risk was also lower in Group V than in Group I, but without statistical significance (adjusted OR 0.407, P > 0.05). The relationship between the post-vitrification cryopreservation period of blastocysts and singleton birth-weight was evaluated by multiple linear regression analysis, as shown in **Table 4**. After correcting for possible confounding factors as described above, no correlation was found between birth-weight and the post-vitrification blastocyst cryopreservation period (P > 0.05).

**Table 3:** Regression analysis of blastocyst cryopreservation period and neonatal birth outcomes.

	Group II	Group III	Group IV	Group V
PTB				
Crude OR (95%CI)	1.693 (0.997–2.877)	**1.796** **(1.009–3.195)**	**1.940** **(1.031–3.654)**	1.380 (0.635–2.998)
Adjusted OR (95%CI)	1.419 (0.820–2.456)	1.530 (0.836–2.800)	1.571 (0.732–3.375)	1.196 (0.255–5.605)
VPTB				
Crude OR (95%CI)	1.361 (0.435–4.260)	1.500 (0.434–5.179)	0.357 (0.04–3.221)	1.164 (0.210–6.443)
Adjusted OR (95%CI)	0.982 (0.291–3.312)	1.480 (0.409–5.363)	0.390 (0.037–4.157)	2.807 (0.127–62.025)
LBW				
Crude OR (95%CI)	1.966 (0.937–4.126)	**2.371** **(1.083–5.193)**	**2.708** **(1.170–6.264)**	2.431 (0.940–6.289)
Adjusted OR (95%CI)	1.760 (0.822–3.766)	**2.376** **(1.058–5.335)**	**3.147** **(1.186–8.352)**	**6.593** **(1.182–36.772)**
VLBW				
Crude OR (95%CI)	0.667 (0.111–4.013)	2.561 (0.513–12.792)	0.713 (0.064–7.918)	NA
Adjusted OR (95%CI)	0.553 (0.083–3.672)	2.275 (0.385–13.456)	0.655 (0.027–15.686)	NA
HBW				
Crude OR (95%CI)	1.043 (0.662–1.643)	**0.482** **(0.262–0.885)**	1.065 (0.594–1.910)	**0.360** **(0.136–0.952)**
Adjusted OR (95%CI)	0.979 (0.604–1.587)	0.429 (0.224–0.821)	1.084 (0.493–2.383)	0.407 (0.058–2.843)
LGA				
Crude OR (95%CI)	1.138 (0.818–1.583)	0.891 (0.608–1.306)	1.419 (0.939–2.147)	0.980 (0.591–1.627)
Adjusted OR (95%CI)	1.105 (0.781–1.564)	0.841 (0.560–1.262)	1.470 (0.882–2.451)	1.176 (0.411–3.366)
SGA				
Crude OR (95%CI)	0.815 (0.385–1.724)	1.087 (0.485–2.435)	0.780 (0.283–2.146)	1.058 (0.359–3.115)
Adjusted OR (95%CI)	0.864 (0.387–1.929)	1.430 (0.595–3.438)	1.179 (0.342–4.065)	3.353 (0.414–27.150)

LBW, low birth-weight; VLBW, very low birth-weight; HBW, high birth-weight; PTB, preterm birth; VPTB, very preterm birth; LGA, large-for-gestational-age; SGA, small-for-gestational-age; OR, odds ratio; CI, confidence interval; NA, not available; The bold values meant there was significant difference. P < 0.05 is considered statistically significant.

**Table 4:** Association between cryopreservation period and singleton birth-weight.

Items	Birth-weight			Gestational age		
	β (95%CI)	Standard error	P	β (95%CI)	Standard error	P
Cryopreservation period						
Group I	Reference			Reference		
Group II	-4.974 (-46.781- 36.833)	21.313	0.816	-0.756 (-1.775- 0.262)	0.519	0.145
Group III	-27.648 (-59.430 - 4.134)	16.202	0.088	-0.674 (-1.448 - 0.101)	0.395	0.088
Group IV	5.389 (-26.283 - 37.061)	16.146	0.739	-0.283 (-1.054 - 0.489)	0.393	0.472
Group V	-6.810 (-57.779 - 44.158)	25.983	0.793	0.068 (-1.173 - 1.310)	0.633	0.914
Maternal age	1.581 (-45.968 - 49.129)	24.240	.948	-.0542 (-1.700 - 0.616)	.591	.359
Maternal age at oocyte	-2.688 (-50.495 - 45.119)	24.371	.912	.0430 (-0.734- 1.595)	.594	.469
Infertility duration (years)	-2.718 (-13.244 - 7.808)	5.366	.613	-.0238 (-0.495 - 0.018)	.131	.069
BMI (kg/m2)	5.825 (-13.244 - 7.808)	8.613	.499	0.190 (-0.221- 0.602)	.210	.365
Endometrial thickness (mm)	-21.975 (-11.070 - 22.721)	13.001	.091	-0.537 (-1.158- 0.084)	.317	.090
Type of infertility (compared with primary)	14.104 (-47.477-3.526)	31.638	.656	0.398 (-1.114-1.910)	.771	.606
Parity (compared with 0)	-7.083 (-47.956-76.164)	18.327	.699	-0.581 (-1.456-0.295)	.446	.194
Fertilization method (compared with IVF)	77.508 (-43.033-28.867)	41.865	.064	-0.272 (-2.272-1.729)	1.020	.790
Ovulation stimulation regimen (compared with GnRHa)	23.063 (-4.613-159.630)	34.354	.502	1.252 (-0.390-2.893)	.837	.135
Endometrial preparation (compared with the natural cycle)	14.194 (-55.116-83.504)	35.333	.688	-1.354 (-3.042-0.334)	.861	.116
Tubal factors	4.497 (-64.808-73.802)	35.331	.899	-1.467 (-3.155-0.221)	.861	.089
Endometriosis	12.052(-66.478-90.581)	40.033	.763	2.094 (0.181-4.007)	.975	.032
Abnormal ovulation	-30.086 (-106.773-46.600)	39.094	.442	-0.288 (-2.157-1.580)	.952	.762
Male factors	-5.573 (-84.170-73.024)	40.068	.889	1.024 (-0.890-2.939)	.976	.294
No of blastocysts transferred	-55.926 (-126.196-14.344)	35.823	.119	-1.876 (-3.588-0.164)	.873	.032
D5 blastocyst transferred	6.510 (-30.146-43.166)	18.687	.728	-0.104 (-.997-0.789)	.455	.819
Good-quality blastocysts transferred	27.394 (-18.285-73.072)	23.286	.240	0.060 (-1.053-1.173)	.567	.916
Offspring sex	88.235 (30.536-145.934)	29.414	.003	-0.589 (-1.995-0.816)	.717	.411

BMI, body mass index; IVF, in vitro fertilization; GnRHa, gonadotropin-releasing hormone agonists. CI, confidence interval; P < 0.05 is considered statistically significant.

## 4 Discussion

The present study analyzed the relationship between the cryopreservation period of vitrified blastocysts and neonatal outcomes. There were no significant differences in neonatal outcomes, including GA and singleton birth-weight, among the groups with different cryopreservation durations. LBW, PTB, SGA, and LGA rates were not significantly different among the groups. In this study, the longest cryopreservation period was approximately 12 years, and the average cryopreservation period in Group V was 5 years; that is, cryopreservation of vitrified blastocysts for 5 years did not affect the birth-weight of singleton newborns.

Early studies have found that with extended cryopreservation, human and mouse embryo post-resuscitation survival rates decrease (27), and chromosomal abnormalities increase. However, some studies indicated that the cryopreservation period did not affect the quality or survival of frozen–thawed embryos (28). Mazur et al. (29) stated that cells can still survive after being frozen in liquid nitrogen for 1000 years because the enzyme activity in the cells is almost completely inhibited in liquid nitrogen and that the cells are in a “stagnant state,” allowing the embryo to maintain its developmental potential for a long time. However, previous studies have focused on slow freezing. With the widespread application of vitrification, the impact of the post-vitrification cryopreservation period on clinical outcomes has attracted attention, but few studies have investigated the impact of the cryopreservation period on neonatal birth-weight, and most previous studies have analyzed the outcomes of long-term cryopreservation in cleavage-stage embryos. For example, Li et al. (30) found that after vitrification of cleavage-stage embryos in open carriers, the GA, birth-weight of singletons, and proportion of boys were not affected by at least 5 years’ cryopreservation. Moreover, some studies included both cleavage and blastocysts (15, 16, 21). For example, Xu et al. (16) found that the post-vitrification cryopreservation period (within 3 years) did not affect the birth-weight of newborns. Another large, single-center study, which included 24,698 patients who underwent whole embryo cryopreservation, showed that, although cryopreservation time could significantly affect the clinical pregnancy and live birth rates, it had no effect on neonatal outcomes (21). However, their study included only patients with cryopreservation periods < 2 years. Additionally, all patients had whole embryos frozen and were eager to undergo transfer earlier, which may have produced some bias. Moreover, the transferred embryos included both cleaved embryos and blastocysts, which may have affected the results. In contrast, we included only frozen–thawed blastocyst transfers in our study.

Our results suggested that the long-term vitrification of blastocysts was relatively safe, similar to the conclusions made in some previous studies (17, 31–33). Wirleitner et al. (31) found that the cryopreservation duration of human blastocysts after vitrification did not affect the viability and developmental potential of the blastocysts and found no significant differences in the embryo implantation, clinical pregnancy, and live birth rate, or neonatal characteristics among the groups. Lee et al. (32) found that storage of vitrified blastocysts for up to 5 years did not affect pregnancy outcomes or neonatal birth-weight. They believe that it is safe for blastocysts to be stored in liquid nitrogen in the form of a nitrogen slurry for a long time after vitrification and that there is no effect on pregnancy and neonatal outcomes. Some studies, including euploid blastocysts that were selected for FET after PGT, found that post-vitrification cryopreservation for up to 3 years had no effect on neonatal birth outcomes (33). In a recent retrospective study involving 6,900 patients, although cryopreservation of blastocysts for more than 6 years significantly reduced the clinical and live birth rates, it did not significantly affect the birth outcome of newborns (17). Nevertheless, Ueno et al. (14) found that, after vitrified blastocysts were stored for 1–97 months, the live birth rate, neonatal birth-weight and proportion of male embryos born were not different from those in two other groups with shorter freezing time. However, the GA of the longest-preserved group was the lowest among the groups. The authors concluded that, although this group had the shortest GA (38.1 ± 1.7 weeks), it had little impact on neonatal health and that the blastocysts could be preserved for a long time post-vitrification. In a recent study involving 31,143 patients, the cryopreservation period did not affect neonatal birth-weight, but low birth-weight was not likely to occur after cryopreservation > 1 year, and the male-to-female ratio in the > 2-year cryopreservation group was significantly lower than that in several groups with a cryopreservation time of < 2 years (34). They suggested that this difference may be because patients in the longest cryopreserved period group were the oldest, and most of the embryos transferred were of poor quality. However, the underlying mechanism is unclear, and further investigation is required.

Considering that the development speed of blastocysts may affect the birth-weight of newborns, we also included the D5 blastocysts transferred as a confounding factor in the multiple regression analysis. Our study is the first to compare the relationship between blastocyst cryopreservation duration and birth-weight considering the blastocyst development speed. Although the proportion of D5 blastocysts transferred differed significantly among the groups, we found that blastocyst development speed had no effect on the singleton birth-weight in multiple linear regression analysis. In the multiple logistic regression analysis, there was no significant correlation between the vitrification preservation period and birth-weight after correcting for possible confounding factors of neonatal birth-weight. However, the LBW incidence in Groups III, IV, and V was significantly higher than that in Group 1 (adjusted OR 2.376, 3.147, and 6.593, P < 0.05, respectively), in contrast to previous studies (17, 32), possibly due to different study conditions. For example, some studies did not adjust for confounding factors that may affect birth-weight (32). Cryopreservation periods also differed across studies. Yan et al. found that the risk of LBW in the group with a cryopreservation time of 5–6 years was 2.19 (range, 0.94–5.07) times that in the group with a cryopreservation time < 3 years, although without statistically significant difference (P = 0.068) (17). Additionally, previous studies have reported that embryo cryopreservation resulted in the risk of increased GA, which may be related to epigenetic modification and changes in the embryo culture environment (35, 36). However, the results of this study showed the prolonged cryopreservation period did not increase the gestational days, even slightly lower in Group III, IV, and V than in Group I (shorter cryopreservation period), which may also be related to the fact that the age of patients in Groups III, IV, and V was higher than that of Group I. Gestational age decreases with increasing age in women, and parity also increases the risk of GA decrease, which may lead to LBW (37). With the extension of the cryopreservation period, the age and parity of the patients increased gradually, which may be one of the reasons. In the future, the correlations among GA, neonatal birth-weight, and cryopreservation period will need to be further analyzed in large-scale studies.

This study had some limitations. First, there may be some selection bias due to the nature of a retrospective study despite the strict inclusion and exclusion criteria. To ensure that patients become pregnant in a short time, high-quality blastocysts were preferentially transferred, which may have created some bias in blastocyst selection. Second, the study sample was relatively small, particularly for long-term frozen embryos. In future, multicenter cooperation should be considered for large-scale data analysis. Moreover, this study did not follow up with children born after FBT; therefore, the relationship between the cryopreservation period and the long-term growth and development of children born after FBT could not be analyzed. However, in this study, we attempted to use strict inclusion criteria and reduce subject heterogeneity as much as possible. During the study period, the embryo culture, freezing, and thawing conditions at our center remained stable, ensuring the stability of this study.

In conclusion, our findings suggested that blastocyst vitrification with open vectors for more than 2 years had no significant effect on birth-weight of singletons born after FBT but may increase the risk of LBW. These data can support patients who wish to postpone embryo transfer because of clinical (e.g., PGT and OHSS) or personal reasons and can assist clinicians and patients in deciding on the cryopreservation period based on risk/benefit analysis. However, future large-scale studies are needed to analyze the impact of long-term cryopreservation on neonatal outcomes and the long-term health of the children born in these procedures.

## Data availability statement

The original contributions presented in the study are included in the article/supplementary material, further inquiries can be directed to the corresponding author/s.

## Ethics statement

The studies involving humans were approved by the Ethics Committee of the Peking Union Medical College Hospital. The studies were conducted in accordance with the local legislation and institutional requirements. The ethics committee/institutional review board waived the requirement of written informed consent for participation from the participants or the participants’ legal guardians/next of kin because This study is a retrospective study.

## Author contributions

XW: Conceptualization, Data curation, Methodology, Writing – original draft, Writing – review & editing, Formal analysis, Software. YX: Data curation, Methodology, Software, Writing – review & editing, Resources. ZS: Conceptualization, Formal analysis, Funding acquisition, Investigation, Methodology, Writing – review & editing. WX: Data curation, Methodology, Resources, Software, Writing – original draft.
